# Georgia Medical Association

**Published:** 1893-03

**Authors:** 


### Editorial.

### GEORGIA MEDICAL ASSOCIATION.

The Georgia Medical Association will hold its annual meeting in Americus, on April 19th, 20th and 21st. This meeting promises to be one of the most valuable and successful meetings yet held. It is the first time the association has met in this section of the State in a number of years, and the hospitality of its people is too well known to need mention at our hands.

The officers of the association are already hard at work preparing the pulminary preliminary of papers and discussions. The voluntary contributions are already very flattering in their promise of valuable material.

They have secured the services of the most efficient medical stenographer to write out the discussions for publication in the transactions, thus insuring an excellent report of all the proceedings.

It is the duty of every physician to attend these meetings, his duty not only to himself but to his fellow-practitioners. To come to them prepared to report some of your interesting cases, to enter into the discussion of those topics in which we all are interested.

There is nothing which brings out the merits or demerits of a paper like discussing them. The friction of ideas is always valuable, it starts us to thinking, as to whether our opinion, which we may have held so long, is the best after all. It starts our ideas in new lines, which may prove valuable to our patrons and ourselves alike. Then it is the place of reunion between old friends. The place where we can shake the hands of those who are close and dear to us but whom we only meet on occasions of this kind.

